# Supplementary material for: Diverse and atypical manifestations of Q fever in a metropolitan city hospital: Emerging role of next-generation sequencing for laboratory diagnosis of Coxiella burnetii
Source: PLoS Negl Trop Dis. 2022 Apr 20;16(4):e0010364. doi: 10.1371/journal.pntd.0010364 (PMC9060374; doi:10.1371/journal.pntd.0010364)
Supplement: S1 Table — (DOCX) [file pntd.0010364.s001.docx]

**S1 Table. Primers and probe for *Coxiella burnetii*** ***IS*1111 gene nested real-time PCR**

| **Primer name** | **Primer sequence (5’→3’)** |
| --- | --- |
| IS1OutF1 (662) | GCGTGGTGATGGAAGCGTGTGGAGGAGCGAACC |
| CoxOUT_R3 (748) | TTGATCAGCGCCGTGCGGCTTTTGACTAAACGATCGC |
| IS1pri_f (500) | CGCAGCACGTCAAACCG |
| IS1IN_R2 (665) | CGCTTCAGCTATCGCCTGC |
| Tqpro_IS1 (506) (probe) | HEX-ATGTCAAA/ZEN/AGTAACAAGAATGATCGTAAC-3IABkFQ |
