# Supplementary material for: Diverse and atypical manifestations of Q fever in a metropolitan city hospital: Emerging role of next-generation sequencing for laboratory diagnosis of Coxiella burnetii
Source: PLoS Negl Trop Dis. 2022 Apr 20;16(4):e0010364. doi: 10.1371/journal.pntd.0010364 (PMC9060374; doi:10.1371/journal.pntd.0010364)
Supplement: S2 Table — (DOCX) [file pntd.0010364.s002.docx]

**S2 Table. Master mix for *Coxiella burnetii* *IS*1111 gene** **nested real-time PCR**

| **Reaction Mix** | **Working concentration** | **Volume (μL)** |
| --- | --- | --- |
| Nuclease-free water | - | 2.6 |
| 2× QuantiNova Probe PCR MM | 1× | 10 |
| IS1OutF1 (5 μM) | 0.05 μM | 0.2 |
| CoxOUT_R3 (5 μM) | 0.05 μM | 0.2 |
| IS1pri_f (20 μM) | 0.5 μM | 0.5 |
| IS1IN_R2 (20 μM) | 0.5 μM | 0.5 |
| Tqpro_IS1 (5 μM) | 1 μM | 1 |
| Sub-total |  | 15 |
| DNA Template |  | 5 |
| Total |  | 20 |
