# Supplementary material for: Diverse and atypical manifestations of Q fever in a metropolitan city hospital: Emerging role of next-generation sequencing for laboratory diagnosis of Coxiella burnetii
Source: PLoS Negl Trop Dis. 2022 Apr 20;16(4):e0010364. doi: 10.1371/journal.pntd.0010364 (PMC9060374; doi:10.1371/journal.pntd.0010364)
Supplement: S3 Table — (DOCX) [file pntd.0010364.s003.docx]

**S3 Table. Cycling profile of *Coxiella burnetii* *IS*1111 gene** **nested real-time PCR**

| **Steps** | | **Temperature** | **Time** | **Acquisition** | **Number of cycles** |
| --- | --- | --- | --- | --- | --- |
| Activation | | 95°C | 2 min | None | 1× |
| First amplification | Denaturation | 95°C | 5 s | None | 20× |
|  | Annealing | 69°C | 30 s | None | 20× |
| Second amplification | Denaturation | 95°C | 5 s | None | 40× |
|  | Annealing | 55°C | 30 s | Single | 40× |
