# Supplementary material for: Diverse and atypical manifestations of Q fever in a metropolitan city hospital: Emerging role of next-generation sequencing for laboratory diagnosis of Coxiella burnetii
Source: PLoS Negl Trop Dis. 2022 Apr 20;16(4):e0010364. doi: 10.1371/journal.pntd.0010364 (PMC9060374; doi:10.1371/journal.pntd.0010364)
Supplement: Attachment — Submitted filename: PNTD-D-22-00084.pdf [file pntd.0010364.s004.pdf]

PLOS Neglected Tropical Diseases

Diverse and atypical manifestations of Q fever in a metropolitan city hospital: emerging role of next-generation sequencing for laboratory diagnosis of Coxiella burnetii, a potential biological warfare agent

--Manuscript Draft--

**Manuscript Number:**	PNTD-D-22-00084
**Full Title:**	Diverse and atypical manifestations of Q fever in a metropolitan city hospital: emerging role of next-generation sequencing for laboratory diagnosis of Coxiella burnetii, a potential biological warfare agent
**Short Title:**	Q fever in metropolitan city hospital
**Article Type:**	Research Article
**Keywords:**	Q fever; Coxiella burnetii; glomerulonephritis; peritonitis; endocarditis; brucellosis; next-generation sequencing
**Abstract:**	Although Q fever has been widely reported in the rural areas of China, there is a paucity of data on the epidemiology and clinical characteristics of this disease in large metropolitan cities. In this study, we profile the epidemiology and clinical manifestations of Q fever from a tertiary hospital in Shenzhen, a Southern Chinese metropolitan city with a large immigrant population from other parts of China. A total of 14 patients were confirmed to have Q fever during a nine-year-and-six-month period, five of whom were retrospectively diagnosed during case review or incidentally picked up because of another research project on unexplained fever without localizing features. Some patients had the typical occupation and/or exposure history, while a few other patients have rare manifestations of Q fever, including one with heart failure and diffuse intracapillary proliferative glomerulonephritis, a patient presenting with a spontaneous bacterial peritonitis-like syndrome, and another one with concomitant Q fever and brucellosis. Using a combination of clinical manifestation, inflammatory marker levels, echocardiographic findings and serological or molecular test results, nine, three and two patients were diagnosed to have acute, chronic and convalescent Q fever, respectively. Seven, five and two patients were diagnosed to have Q fever by serological test, nested real-time PCR and next-generation sequencing respectively. Due to its diverse and atypical manifestations not recognized by clinicians, the disease was often self-limiting or has responded to empirical doxycycline prescribed for other purposes, and a lack of laboratory support in some hospitals, the incidence of Q fever is likely to be underestimated. Next-generation sequencing is becoming an important diagnostic modality for culture-negative infections, particularly those that the physicians fail to recognize clinically, such as Q fever.
**Additional Information:**	
**Question**	**Response**
**Financial Disclosure**<br><br>Enter a financial disclosure statement that describes the sources of funding for the work included in this submission. Review the submission guidelines for detailed requirements. View published research articles from *PLOS NTDs* for specific examples.<br><br>This statement is required for submission and **will appear in the published article** if the submission is accepted. Please make sure it is accurate.	This study was partly supported by Sanming Project of Medicine in Shenzhen, China (SZSM201911014, http://wjw.sz.gov.cn/ztzl/smgc/), and FX received the award for this study. The funders had no role in study design, data collection and analysis, decision to publish, or preparation of the manuscript.

Powered by Editorial Manager® and ProduXion Manager® from Aries Systems Corporation

**Unfunded studies**<br><br>Enter: *The author(s) received no specific funding for this work.*<br><br>**Funded studies**<br><br>Enter a statement with the following details:<br>• Initials of the authors who received each award<br>• Grant numbers awarded to each author<br>• The full name of each funder<br>• URL of each funder website<br>• Did the sponsors or funders play any role in the study design, data collection and analysis, decision to publish, or preparation of the manuscript?<br>• **NO** - Include this sentence at the end of your statement: *The funders had no role in study design, data collection and analysis, decision to publish, or preparation of the manuscript.*<br>• **YES** - Specify the role(s) played.<br><br>* typeset	
**Competing Interests**<br><br>Use the instructions below to enter a competing interest statement for this submission. On behalf of all authors, disclose any competing interests that could be perceived to bias this work—acknowledging all financial support and any other relevant financial or non-financial competing interests.<br><br>This statement **will appear in the published article** if the submission is accepted. Please make sure it is accurate. View published research articles from *PLOS NTDs* for specific examples.	The authors have declared that no competing interests exist.

**NO authors have competing interests**<br><br>Enter: *The authors have declared that no competing interests exist.*<br><br>**Authors with competing interests**<br><br>Enter competing interest details beginning with this statement:<br><br>*I have read the journal's policy and the authors of this manuscript have the following competing interests: [insert competing interests here]*<br><br>* typeset<br><br>This statement is required for submission and **will appear in the published article** if the submission is accepted. Please make sure it is accurate and that any funding sources listed in your Funding Information later in the submission form are also declared in your Financial Disclosure statement.	
**Data Availability**<br><br>Authors are required to make all data underlying the findings described fully available, without restriction, and from the time of publication. PLOS allows rare exceptions to address legal and ethical concerns. See the PLOS Data Policy and FAQ for detailed information.	Yes - all data are fully available without restriction

A Data Availability Statement describing where the data can be found is required at submission. Your answers to this question constitute the Data Availability Statement and **will be published in the article**, if accepted.<br><br>**Important:** Stating 'data available on request from the author' is not sufficient. If your data are only available upon request, select 'No' for the first question and explain your exceptional situation in the text box.<br><br>Do the authors confirm that all data underlying the findings described in their manuscript are fully available without restriction?	
**Describe where the data may be found in full sentences. If you are copying our sample text, replace any instances of XXX with the appropriate details.**<br><br>• If the data are **held or will be held in a public repository**, include URLs, accession numbers or DOIs. If this information will only be available after acceptance, indicate this by ticking the box below. For example: *All XXX files are available from the XXX database (accession number(s) XXX, XXX.*).<br>• If the data are all contained **within the manuscript and/or Supporting Information files**, enter the following: *All relevant data are within the manuscript and its Supporting Information files.*<br>• If neither of these applies but you are able to provide **details of access elsewhere**, with or without limitations, please do so. For example:	All relevant data are within the manuscript and its Supporting Information files.

Data cannot be shared publicly because of [XXX]. Data are available from the XXX Institutional Data Access / Ethics Committee (contact via XXX) for researchers who meet the criteria for access to confidential data.<br><br>*The data underlying the results presented in the study are available from (include the name of the third party and contact information or URL).*<br>• This text is appropriate if the data are owned by a third party and authors do not have permission to share the data.<br><br>* typeset	
Additional data availability information:	

Powered by Editorial Manager® and ProduXion Manager® from Aries Systems Corporation

Diverse and atypical manifestations of Q fever in a metropolitan city hospital: emerging role of next-generation sequencing for laboratory diagnosis of *Coxiella burnetii*, a potential biological warfare agent

Short title: Q fever in metropolitan city hospital

Fanfan Xing[1], Haiyan Ye[1], Chaowen Deng[1], Linlin Sun[1], Yanfei Yuan[1], Qianyun Lu[1], Jin Yang[1], Simon K.F. Lo[1], Ruiping Zhang[2], Jasper F.W. Chan[3], Susanna K.P. Lau[3]*, Patrick C.Y. Woo[3]*

[1]Department of Clinical Microbiology and Infection Control, The University of Hong Kong - Shenzhen Hospital, Shenzhen, Guangdong, China

[2]Department of Pathology, The University of Hong Kong - Shenzhen Hospital, Shenzhen, Guangdong, China

[3]Department of Microbiology, Li Ka Shing Faculty of Medicine, The University of Hong Kong, Hong Kong, China

*Corresponding authors

E-mail: pcywoo@hku.hk (PCYW); skplau@hku.hk (SKPL)

Abstract

Although Q fever has been widely reported in the rural areas of China, there is a paucity of data on the epidemiology and clinical characteristics of this disease in large metropolitan cities. In this study, we profile the epidemiology and clinical manifestations of Q fever from a tertiary hospital in Shenzhen, a Southern Chinese metropolitan city with a large immigrant population from other parts of China. A total of 14 patients were confirmed to have Q fever during a nine-year-and-six-month period, five of whom were retrospectively diagnosed during case review or incidentally picked up because of another research project on unexplained fever without localizing features. Some patients had the typical occupation and/or exposure history, while a few other patients have rare manifestations of Q fever, including one with heart failure and diffuse intracapillary proliferative glomerulonephritis, a patient presenting with a spontaneous bacterial peritonitis-like syndrome, and another one with concomitant Q fever and brucellosis. Using a combination of clinical manifestation, inflammatory marker levels, echocardiographic findings and serological or molecular test results, nine, three and two patients were diagnosed to have acute, chronic and convalescent Q fever, respectively. Seven, five and two patients were diagnosed to have Q fever by serological test, nested real-time PCR and next-generation sequencing respectively. Due to its diverse and atypical manifestations not recognized by clinicians, the disease was often self-limiting or has responded to empirical doxycycline prescribed for other purposes, and a lack of laboratory support in some hospitals, the incidence of Q fever is likely to be underestimated. Next-generation sequencing is becoming an important diagnostic modality for culture-negative infections, particularly those that the physicians fail to recognize clinically, such as Q fever.

Author summary

We describe the epidemiology and clinical manifestations of Q fever from a tertiary hospital in Shenzhen, a Southern Chinese metropolitan city in China. A total of 14 patients were confirmed to have Q fever during this study period. Notably, five of them were retrospectively diagnosed during case review or incidentally picked up because of another research project on patients with unexplained fever. Interestingly, some patients had rare manifestations of Q fever, such as heart failure and diffuse intracapillary proliferative glomerulonephritis and spontaneous bacterial peritonitis. One patient had concomitant Q fever and brucellosis. Half of the patients were diagnosed by traditional serological test, while the other half by PCR or next-generation sequencing. Clinicians should have a high index of suspicion of Q fever because of its diverse and atypical manifestations. The incidence of Q fever is likely to be underestimated. Next-generation sequencing is becoming increasingly important for diagnosis of culture-negative infections.

Introduction

Q fever is a zoonotic infection caused by a pleomorphic intracellular bacterium, *Coxiella burnetii*. Domestic animals, mainly sheep, goats and cattle, are the major source for human infection [1], with the bacterium present in the faeces, urine, milk and placenta of the infected animals. In addition, *C. burnetii* can also be found in many ~~other~~ wild and domestic animals such as horses, dogs, pigs, some birds, etc. [2]. The major route of transmission of *C. burnetii* to human is through inhalation of contaminated aerosols and dust particles as well as handling and ingestion of infected meat and milk. Therefore, those who are in close contact with the animals, such as farmers, abattoir workers and veterinarians are at highest risk. Clinical presentation of Q fever can be acute or chronic. The acute form of the disease usually presents as a self-limited non-specific febrile illness or atypical pneumonia, whereas the manifestation of the chronic form is more variable, although the commonest one is endocarditis. Notably, Q fever has become a notifiable disease in the United States since 1999 due to its potential as a biological warfare agent [3]. Traditionally, Q fever is diagnosed in the laboratory using serological test by detection of antibodies. Recently, molecular tests such as polymerase chain reaction (PCR) amplification of specific targets have also been employed for more rapid diagnosis of this condition [4].

Although Q fever has been widely reported in the rural areas of China [5], there is a paucity of data on the epidemiology and clinical characteristics of this disease in large metropolitan cities. Since it is relatively uncommon in modern cities, diagnosis is often difficult as most clinicians may be unaware of the diverse manifestations of the disease. Often, the disease may be treated without noticing the diagnosis through the prescription of empirical doxycycline for atypical pneumonia or fever without localizing features. In this study, we profile the epidemiology and clinical manifestations of Q fever from a tertiary hospital in Shenzhen, a Southern Chinese metropolitan city with a large immigrant population from other parts of

China. In addition, the use of next-generation sequencing (NGS), the state-of-the-art and emerging technology in clinical microbiology, for laboratory diagnosis of Q fever as well as other culture-negative infectious disease syndromes is also discussed.

Materials and methods

Ethical statement

Ethics approval for this study (No. [2021]161) was provided by the Institutional Review Board of The University of Hong Kong - Shenzhen Hospital.

Patients

This was a retrospective study conducted over a nine-year-and-six-month period (1 July 2012 to 31 December 2021) in The University of Hong Kong - Shenzhen Hospital. This 1,400-bed multi-specialty hospital was established in 2012 and provides primary to tertiary medical services to the residents of Shenzhen city in both inpatient and outpatient settings. Shenzhen is a Special Economic Zone with an estimated population of nearly 18 million people including a large migrant population from other regions in China. Geographically, it is located in the Guangdong Province, immediately north to Hong Kong. Affected by the policy of the government in mainland China, Shenzhen has been one of the fastest growing cities in the world during the 1990s. The clinical details, laboratory data and radiological findings of all patients with Q fever were retrieved from the hospital electronic record system and analysed. The diagnosis of acute, chronic and convalescent Q fever was made based on a combination of clinical presentation, inflammatory marker levels, echocardiographic findings and serological or molecular test results. Endocarditis was diagnosed using modified Duke's criteria [6].

Indirect immunofluorescence assay

Q fever serology was performed in our laboratory since September 2020 using the indirect immunofluorescent assay (Focus Diagnostics, California, USA) for detection of human IgM antibodies to *C. burnetii* by a 2-stage "sandwich" principle, in which the wells of the slide was coated with *C. burnetii* phase I/II antigen and the presence of IgM detected with fluorescein-labeled antibody to IgM. The test was performed and results interpreted according to manufacturer's instructions. A serum titer of ≥1:16 to both phase I and phase II antigens strongly suggests recent *C. burnetii* infection, while that of <1:16 to both phase I and phase II antigens argues against recent *C. burnetii* infection. During acute infection, the IgM titers to phase II antigen are greater than those to phase I antigen; whereas during chronic infection or convalescent phase, the IgM titers to phase I antigen are ~~great~~ than or equal to phase II antigen. Detection of IgG antibodies was not performed because of budget limitations.

Nested real-time PCR

Nested real-time PCR for *C. burnetii* was performed in our laboratory since August 2021 by targeting the transposon-like repetitive region, *IS1111* gene. Total nucleic acid was extracted from 300 μL of plasma using the MagaBio plus Virus DNA/RNA Purification Kit III (BIOER, Hangzhou, China). The nucleic acid was eluted in 60 μL of RNase-free water and was used as the template for nested real-time PCR. The primers and probe sequences of the nested real-time PCR assay were synthesized by BGI (Beijing, China) (S1 Table). Real-time PCR was performed using QuantiNova Probe PCR Kit (Qiagen) in a QuantStudio™ 5 Real-Time PCR Instrument (ABI, Singapore). The master mix and cycling conditions are shown in S2 and S3 Tables.

Next-generation sequencing

Ethylene Diamine Tetraacetic Acid (EDTA)-treated blood was collected from the patients and sent to the BGI PathoGenesis Pharmaceutical Technology Co., Ltd (Shenzhen, China) for NGS analysis of pathogenic microorganisms.

Results

Clinical characteristics

A total of 14 patients were confirmed to have Q fever during the study period (Table 1). Twelve patients were males and two were females. The median age was 46.5 (range 20-65). Three had high risk occupations (chef in case 6 and farmers in case 10 and 11). Four (case 2, 6, 10 and 11) had clear histories of recent exposure to goat, sheep or cattle and 4 others (case 3, 8, 9 and 12) ~~have~~ recent visit to ~~the~~ rural environment. The remaining 6 patients (case 1, 4, 5, 7, 13 and 14) denied any recent contact with livestock, although case 1 had recent unprotected sexual intercourse, which has been reported to be a possible route of *C. burnetii* transmission [7]. The median interval between disease onset and hospital admission was 10 (range 6-90) days and that between hospital admission and confirmation of the diagnosis of Q fever was 10.5 (range 3-600) days. All the 14 patients presented with fever and non-specific symptoms, although case 2 and 3 had very severe headache and were admitted to the neurology unit as suspected meningitis. Case 6 presented with symptoms of heart failure and glomerulonephritis (Fig 1) and case 9 presented with a spontaneous bacterial peritonitis-like syndrome (Fig 2). Four (case 1, 2, 6 and 9) and 9 (case 1, 2, 3, 4, 6, 7, 8, 10 and 14) patients had hepatomegaly and splenomegaly, respectively. Using a combination of clinical manifestation, inflammatory marker levels, echocardiographic findings and serological or molecular test results, 9 (case 1, 2, 3, 4, 5, 7, 8, 11 and 12), 3 (case 6, 9 and 14) and 2 (case 10 and 13) patients were diagnosed to have acute, chronic and convalescent Q fever, respectively. All the 14 patients survived. For ~~the~~ 10 patients (case 1, 2, 3, 4, 5, 6, 8, 9, 12 and 13) who had fever on admission, the median time to defervescence was 3.5 (range 1-7) days.

Table 1. Demographic and clinical characteristics of patients in the present cohort

Patient No.	Year of diagnosis	Sex/Age	Occupation	Exposure history	Interval between disease onset to hospital visit (days)	Interval between hospitalization and diagnosis (days)	Form of Q fever	Underlying disease	Clinical manifestation	Chest radiographic finding	Abdominal imaging finding	Echocardiography	Days from antibiotic treatment to defervescence
1	2014	M/27	Docker	Unprotected sexual exposure	11	8	Acute	None	Fever, chills, weakness, arthralgia, myalgia, relative bradycardia, hepatomegaly, splenomegaly	None	Gallbladder wall thickening, hepatomegaly, splenomegaly	Normal	6
2	2019	M/37	Engineer	Dog, goat meat, rural environment	6	3	Acute	Hypertension	Fever, chills, night sweats, weakness, headache, arthralgia, myalgia, nausea, vomiting, abdominal pain, lower back pain, cough, conjunctival congestion, relative bradycardia, jaundice, hepatomegaly, splenomegaly	None	Hepatomegaly, splenomegaly, kidney stone	Normal	2
3	2019	M/65	Headmaster	Guinea pigs, hens, rural environment	7	5	Acute	Hypertension, secondary hypothyroidism	Fever, weakness, headache, arthralgia, myalgia, conjunctival congestion, relative bradycardia, splenomegaly	Bilateral patchy infiltrates and atelectasis	Splenomegaly	Sclerosis of aortic valves	3
4	2020	M/20	Student	Unclean food	9	600	Acute	None	Fever, chills and rigors, splenomegaly	Normal	Splenomegaly	Normal	1
5	2020	M/40	Unemployed	Dogs, rabbits	6	570	Acute	None	Fever, skin rash, chills, general pain	Multiple pulmonary bullae	No abnormality	Normal	3
6	2020	M/62	Chef	Livestock, rural environment	7	12	Chronic	Hypertension, congestive heart failure	Fever, facial puffiness, lower limb edema, night sweats, weakness, abdominal pain, cough, dyspnea, relative bradycardia, lymphadenopathy, hepatomegaly, splenomegaly	Bilateral patchy infiltrates and pleural effusion	Splenomegaly, enlarged bilateral kidneys	Thickening of mitral and tricuspid valves and chordae tendineae; aortic valves stenosis with insufficiency and suspected abscess or hematoma; pericardial effusion	4
7	2020	M/35	Clerk	None	14	510	Acute	None	Fever, dizziness	Inflammation in bilateral lower lung and the left lingual lobe	Cholecystitis, ascites, left kidney stone, splenomegaly	Normal	Still afebrile when discharged

8	2021	M/44	Clerk	Lizards, tortoise, fresh water fish, crickets, bovine placenta; rural environment	6	300	Acute	None	Fever, headache, nausea and vomiting	Micronodules seen in the left lung, lymph nodes or inflammatory granulomas suspected	Splenomegaly	Normal	2
9	2021	M/35	Company manager	Rural environment	10	6	Chronic	Fatty liver	Fever, chills, weakness, abdominal pain, relative bradycardia, hepatomegaly	Bilateral pleural effusion and atelectasis	Gallbladder wall thickening, hepatomegaly, fatty liver, thickened capsule of bilateral kidney, peritonitis	Normal	7
10	2021	F/49	Farmer	Goat	20	9	Convalescent	Hypertension	Fever[a], night sweats, arthralgia, myalgia, splenomegaly	None	Liver cyst, splenomegaly	Enlargement of left atrium	-
11	2021	M/50	Farmer	Goat	90	30	Acute	None	Fever[a], night sweats, arthralgia, low back pain	None	Inflammation of terminal ileum	Diastolic dysfunction of left ventricle	-
12	2021	M/52	Government servant	Cat, rural environment	21	8	Acute	Hypertension, diabetes mellitus, gout	Fever, weakness, rash, chest pain, lymphadenopathy	Bilateral consolidation and pleural effusion	Thickened capsule of bilateral kidneys	Regurgitation of mitral and tricuspid valves; pericardial effusion	4
13	2021	M/56	Unemployed	None	51	45	Convalescent	Chronic obstructive pulmonary disease	Fever, weakness, low back pain, relative bradycardia	None	None	Normal	5
14	2021	F/56	Retired clerk	Dog	10	4	Chronic	Hypertension	Fever[a], chills, headache, splenomegaly	None	Cholecystectomy, splenomegaly	Vegetation of aortic valves; pericardial effusion	-

[a]These patients became afebrile before admission and commencement of antibiotic.

Fig 1. Computed tomography of the thorax and abdomen and histology of renal biopsy for Case 6.

(A) Bilateral diffuse interstitial infiltrates pleural effusion. (B) Bilateral pleural effusion and mediastinal lymphadenopathy (arrow). (C) Hepatosplenomegaly and ascites. (D) Symmetrically enlarged kidneys. (E) Diffuse intracapillary hyperplasia in the glomerulus with neutrophil infiltration in the capillary lumen, and mild proliferation of mesangial cells and stroma in focal segments of the glomerulus (PAS×400). (F) Focal renal interstitial fibrosis and edema with neutrophil, lymphocyte and plasmacyte infiltration (H&E×200). (G) Granular C3 deposition in the capillary wall and mesangial regions on immunofluorescent staining (×200).

Fig 2. Computed tomography of the abdomen for Case 9.

(A) Plain film showing peritonitis (arrowhead) and thickened capsule of the left kidney (arrow). (B) Contrast-enhanced image (arterial phase) showing peritonitis (arrowhead) and thickened capsule of the right kidney (arrow).

Laboratory findings

The laboratory findings of ~~the~~ 14 patients with Q fever in the present cohort are summarized in Table 2. Three of the 10 patients (case 1, 9 and 12) had increased peripheral white cell count and neutrophilia. Five patients (case 2, 3, 5, 6 and 10) had moderate thrombocytopenia. Twelve (case 1, 2, 3, 4, 5, 7, 8, 9, 10, 12, 13 and 14) had mildly to moderately elevated liver parenchymal enzymes. The median (range) serum alanine transaminase and aspartate transaminase levels were 103.2 (11.1-154) U/L and 54.9 (25-167.9) U/L respectively. The median erythrocyte sedimentation rate (ESR) was 28 (range 5-111) mm/hour, with 6 patients (case 1, 4, 5, 6, 7 and 11) having moderately raised ESR and one patient (case 12) with an ESR of >100 mm/hour. The median C-reactive protein (CRP) was 87.5 (range 0.45-219.2) mg/L with 13 patients having elevated CRP. The median activated partial thromboplastin time

(aPTT) was 46.1 (range 33.9-82.3) seconds, with 10 patients (case 1, 4, 5, 6, 7, 8, 9, 12, 13 and 14) having prolonged aPTT. Lupus anticoagulant was checked in 9 patients and 6 (case 1, 6, 7, 9, 12, and 14) were detected.

Table 2. Laboratory findings of patients in the present cohort

Patient No.	WBC ($\times 10^9$/L)	Neutrophil ($\times 10^9$/L)	Platelet ($\times 10^9$/L)	ALT (U/L)	AST (U/L)	Tbil (mmol/L)	ESR (mm/h)	CRP (mg/L)	PT (s)	aPTT (s)	Lupus anticoagulant	Anti-cardiolipin IgM (U/mL)	Anti-cardiolipin IgG (U/mL)	Anti-MPO-IgG (RU/mL)	Anti-PR3-IgG (RU/mL)	RF (U/mL)	Brucella Ab	Diagnostic test for Q fever
**1**	10.9	7.9	366	154	42	15.4	57	86	14.2	54	Detected	213	84.4	26.1	46.2	17	Negative	CF: phase II 1:640 IFA IgM: phase II 1:800
**2**	4.6	3	68	107.5	100.7	53.1	17	179.5	14.4	39.4	Not done	Not done	Not done	Not done	Not done	Not done	Negative	NGS 211 sequences detected
**3**	5.5	4.2	114	46.6	30.9	15.2	17	54	16.7	36.1	Not done	<2	<2	2.05	<2	Not done	Negative	NGS 1021 sequences detected
**4**	5.38	4.16	196	110.6	51.9	14	29	68.61	14	45	Not done	Not done	Not done	5.45	3.01	Not done	Not done	Nested real-time PCR positive
**5**	3.75	2.87	103	124.6	139	10.7	40	98.57	13.7	46.3	Not done	Not done	Not done	Not done	Not done	8.6	Not done	Nested real-time PCR positive
**6**	7	4.6	94	11.1	25	12.7	33	32	13.7	49.8	Detected	7.89	3.67	<2	4.71	43.9	Not done	IFA IgM: phase I > 1:8192, phase II 1:512
**7**	8.54	5.12	178	86.7	56.4	26.4	51	122.91	16.8	51.9	Detected	> 800	> 800	76.3	> 800	21.1	Negative	Nested real-time PCR positive
**8**	6.64	4.91	220	138.4	95	30.9	20	89.05	14.4	45.9	Not detected	Negative	Negative	Not done	Not done	Not done	Negative	Nested real-time PCR positive
**9**	19	15.3	290	152.5	88	16.5	22	124.9	14.9	60.9	Detected	88.2	>480	10.9	25.8	18.9	Not done	IFA IgM: phase I 1:1024, phase II 1:1024
**10**	3.5	2.3	137	76.7	53.4	7.4	5	0.45	12.4	33.9	Not detected	Negative	Negative	Not done	Not done	48.1	Negative	IFA IgM: phase I 1:128, phase II 1:64
**11**	5.72	4.55	158	33	29.5	5	28	12.1	13.6	36.6	Not detected	Negative	Negative	Not done	Not done	8.8	Negative	IFA IgM: phase I negative, phase II 1:64
**12**	13.1	11.2	281	98.8	75.9	26.9	111	219.2	15.5	82.3	Detected	11.3	99.4	11.8	35.7	12.1	Negative	IFA IgM: phase I 1:2048, phase II 1:8192
**13**	6.5	3.4	410	52.4	42.1	5.2	7	40.7	12.5	41.1	Not done	<2	<2	<2	2.07	N/A	1:200	IFA IgM: phase I 1:128; phase II 1:16
**14**	3.3	2.1	151	144.8	167.9	5.3	Not done	143.6	12.5	55.9	Detected	293.2	34.8	4.98	16.6	10.5	Negative	IFA IgM: phase I and II not detected, nested

																		real-time PCR positive

Abbreviation: PCR: polymerase chain reaction; NGS: next-generation sequencing; IFA: immunofluorescence assay; ALT: alanine transaminase; AST: aspartate transaminase; Tbil: total bilirubin; ESR: erythrocyte sedimentation rate; CRP: C-reactive protein; PT: prothrombin time; aPTT: activated partial thromboplastin time; anti-MPO-IgG: anti-myeloperoxidase-IgG; anti-PR3-IgG: anti-proteinase 3-IgG; RF, rheumatoid factor.

Echocardiography findings and endocarditis

Echocardiography was performed in all of the 14 patients, with 6 of them showing abnormal findings (Table 1). According to the modified Duke's criteria, 2 patients (case 6 and 14) fulfilled the criteria for infective endocarditis.

Microbiological findings and laboratory diagnosis of Q fever

Seven patients (case 1, 6, 9, 10, 11, 12 and 13) were diagnosed to have Q fever by positive serological test (Table 2). Five patients (case 4, 5, 7, 8 and 14) were diagnosed by positive nested real-time PCR and two (case 2 and 3) were diagnosed by NGS. Ten patients had brucella serology performed and ~~was positive in one~~ (case 13).

Discussion

In this study, we describe the diverse and some atypical manifestations of Q fever in a densely populated metropolitan city. In the present cohort, some patients had the typical occupation, exposure history and manifestation. For example, cases 10 and 11 were a couple and they were farmers with clear contact history with goats. On the other hand, a few other patients ~~have~~ atypical and rare manifestations of Q fever. Case 9 was a 35-year-old man with underlying fatty liver who presented with fever, chills and abdominal pain. Although the clinical diagnosis was spontaneous bacterial peritonitis, chest radiograph revealed bilateral pleural effusion and atelectasis and contrast computed tomography (CT) of the abdomen showed abdominal effusion, thickening of parietal peritoneum and bilateral renal capsules (Fig 2). In addition, he had prolonged ~~aPTT~~ at 60.9 seconds and he failed to respond to empirical intravenous piperacillin-tazobactam for the treatment of spontaneous bacterial peritonitis. Although trans-esophageal echocardiography did not show any vegetation, Q fever serology

was performed and revealed high titers (both ≥1:1024) of IgM to both phase I and phase II antigens. In the literature, only one other case of Q fever with a spontaneous bacterial peritonitis-like syndrome was reported [8]. In that 55-year-old man with underlying type 2 diabetes mellitus, he presented with fever and chills for 20 days but there was no abdominal pain. Only diffuse abdominal fullness without tenderness was observed during physical examination. Similar to our patient, he also had prolonged aPTT of 74.5 seconds and mildly deranged liver function test. CT of the abdomen did not show any ascites but gallium scan revealed hepatomegaly with diffuse uptake in the abdomen, suggestive of peritonitis or peritoneum carcinomatosis. Q fever serology subsequently showed high titers (both ≥1:2560) of IgG and IgM to phase II antigens. In addition to this case 9 of Q fever presenting as spontaneous bacterial peritonitis, the manifestation of case 6 was also uncommon. Case 6 was a 62-year-old man with underlying hypertension and congestive heart failure who presented with facial puffiness and bilateral lower limb swelling for 5 days without fever. Serum creatinine was elevated and on increasing trend and there was hypoalbuminemia and microscopic hematuria. CT of the thorax and abdomen showed interstitial pulmonary edema, pericardial and bilateral pleural effusion, mediastinal lymphadenopathy, bilateral enlarged kidneys and ascites (Fig 1A-D). Q fever serology showed that the titers of IgM to phase I and phase II antigens were 1:8192 and 1:512 respectively. Histological examination of the renal biopsy revealed diffuse intracapillary proliferative glomerulonephritis (Fig 1E-G), which has been reported only once as a complication of Q fever [9]. Other reported cases of glomerulonephritis associated with Q fever were mainly focal and segmental proliferative glomerulonephritis, mesangioproliferative glomerulonephritis, mesangiocapillary glomerulonephritis and membranoproliferative glomerulonephritis [9-12]. In our patient, the glomerulonephritis and renal function responded promptly to doxycycline treatment of the Q fever.

The incidence of Q fever is underestimated. Failure to make a diagnosis of Q fever is mainly due to the difficulty for the clinician to recognize the disease or lack of laboratory support to confirm the diagnosis. In modern cities where farms are not commonly found and the incidence of Q fever low, doctors are unfamiliar with the diverse presentations of this infection. Moreover, the disease is often self-limited or if presented as atypical pneumonia, it may be treated empirically with doxycycline without confirming the microbiological diagnosis through ordering the appropriate laboratory tests. As illustrated in the present cohort, case 13 was a 56-year-old man presented with fever and back pain. As *Brucella melitensis* was isolated from the patient's blood culture and brucella serology was also positive, the patient was treated with doxycycline and gentamicin for one week followed by doxycycline for five more weeks. The patient responded and was discharged uneventfully. It was only during case review one and a half months later that the diagnosis of Q fever was also suspected. The serum of the patient was retrieved and Q fever serology showed that the titers of IgM to phase I and phase II antigens were 1:128 and 1:16 respectively. In fact, co-infection of *C. burnetii* and *Brucella* species has only been reported once in the literature [13]. In that case, the patient was a 30-year-old agricultural worker who presented with fever and non-specific symptoms. He worked in a sheep farm and has consumed unpasteurized dairy products of sheep origin in Bosnia and Herzegovina. Similar to our case 13, blood culture was positive for *B. melitensis* and brucella serology was also positive. In addition, *C. burnetii* phase II IgM/IgG titers were 1:50 and 1:1024, respectively, confirming the co-infection. As the animal source of these two bacteria are common, we speculate that *C. burnetii* and *Brucella* co-infection is also under reported, as patients who are treated ~~with~~ brucellosis would have their Q fever treated automatically. In addition to case 13, it is of note that four other patients (case 4, 5, 7 and 8) were clinically diagnosed to have typhus-like illness during their admissions, although none of them was laboratory confirmed. Hence, doxycycline was empirically prescribed and they responded

promptly. Their diagnosis of Q fever was only incidentally confirmed by real-time quantitative PCR when they were investigated retrospectively for unexplained fever without localizing features in another research project. As for the lack for laboratory support, some microbiology laboratories are not equipped with tests for Q fever. For example, for the laboratory in our hospital, serology test was only available since late 2020. This is indeed the reason why 70% of the Q fever cases in the present cohort were made since this time. For case 1 which the diagnosis was made in 2014, the laboratory test was actually carried out in Hong Kong when the diagnosis of Q fever was suspected despite there was no obvious exposure histories to animals.

NGS is becoming an important diagnostic modality for culture-negative infections, particularly those that the physicians fail to recognize clinically. When NGS technologies first appeared in the market, they were mainly used for genome sequencing. With the advancement of sequencing chemistries and computational capacity, NGS technologies have matured into clinical applications in ~~the~~ recent years [14]. In the clinical setting for infectious diseases, NGS is used most often for patients who have fever without localizing features or culture-negative infections. We have recently reported its application in fungal diagnosis as well as confirming the first case of listeria meningitis in a patient with autoantibody against interferon gamma and another one with *Mycobacterium marinum* infection [15-17]. In the present cohort, case 2 and 3 both presented with fever and severe headache and were admitted to the neurology unit as suspected meningitis. Lumbar puncture was performed but analysis of the cerebrospinal fluid was negative. At that time, Q fever serology and real-time PCR test were not yet available in our hospital. Hence, blood samples of the patients were sent for NGS, which revealed 211 and 1021 sequence reads of *C. burnetii* respectively, confirming the diagnosis of Q fever. In our setting, the NGS was performed in a private laboratory with the cost of RMB 4,500 (~698 USD) per sample and the turn-around-time for these two cases were two days, making ~~the~~ use

of this robust technology pragmatic and affordable in ~~the~~ clinical setting. It is of note that Q fever has been diagnosed a few times using NGS in the literature [18-20], including a recent outbreak in southern China [19]. In that outbreak, plasma samples from 138 out of 2382 patients who had fever of unknown source were tested positive for *C. burnetii* sequences by NGS and the outbreak was finally traced to goats and cattle in a slaughterhouse [19]. With its low equipment cost, short turn-around-time and portable size, the recent invention of the Oxford Nanopore Technologies' MinION device and further improvement of its sequencing accuracy will make the use of NGS within clinical microbiology laboratories feasible in the near future.

Acknowledgments

We are grateful to the staff at the Department of Clinical Microbiology and Infection Control, the Department of Pathology, the Department of Medical Imaging, The University of Hong Kong-Shenzhen Hospital, and the Department of Microbiology, The University of Hong Kong for facilitation of the study.

References

1. Guatteo R, Seegers H, Taurel AF, Joly A, Beaudeau F. Prevalence of Coxiella burnetii infection in domestic ruminants: a critical review. Vet Microbiol. 2011 Apr 21;149(1-2):1-16. doi: 10.1016/j.vetmic.2010.10.007.
2. Cutler SJ, Bouzid M, Cutler RR. Q fever. J Infect. 2007 Apr;54(4):313-8. doi: 10.1016/j.jinf.2006.10.048.
3. Select Agents and Toxins List. HHS and USDA select agents and toxins [cited 12 Nov 2021]. In: Federal Select Agent Program [Internet]. Federal Select Agent Program 2021. Available from: https://www.selectagents.gov/sat/list.htm.

4. Fenollar F, Fournier PE, Raoult D. Molecular detection of Coxiella burnetii in the sera of patients with Q fever endocarditis or vascular infection. J Clin Microbiol. 2004 Nov;42(11):4919-24. doi: 10.1128/JCM.42.11.4919-4924.2004.
5. El-Mahallawy HS, Lu G, Kelly P, Xu D, Li Y, Fan W, et al. Q fever in China: a systematic review, 1989-2013. Epidemiol Infect. 2015 Mar;143(4):673-81. doi: 10.1017/S0950268814002593.
6. Fournier PE, Casalta JP, Habib G, Messana T, Raoult D. Modification of the diagnostic criteria proposed by the Duke Endocarditis Service to permit improved diagnosis of Q fever endocarditis. Am J Med. 1996 Jun;100(6):629-33. doi: 10.1016/s0002-9343(96)00040-x.
7. Milazzo A, Hall R, Storm PA, Harris RJ, Winslow W, Marmion BP. Sexually transmitted Q fever. Clin Infect Dis. 2001 Aug 1;33(3):399-402. doi: 10.1086/321878.
8. Chang K, Ko WC, Li BF, Liu PY, Chiu NT. Diffuse abdominal uptake mimicking peritonitis in gallium inflammatory scan: an unusual feature of acute Q fever. Kaohsiung J Med Sci. 2005 Nov;21(11):522-6. doi: 10.1016/S1607-551X(09)70161-5.
9. Perez-Fontan M, Huarte E, Tellez A, Rodriguez-Carmona A, Picazo ML, Martinez-Ara J. Glomerular nephropathy associated with chronic Q fever. Am J Kidney Dis. 1988 Apr;11(4):298-306. doi: 10.1016/s0272-6386(88)80134-3.
10. Tolosa-Vilella C, Rodríguez-Jornet A, Font-Rocabanyera J, Andreu-Navarro X. Mesangioproliferative glomerulonephritis and antibodies to phospholipids in a patient with acute Q fever: case report. Clin Infect Dis. 1995 Jul;21(1):196-8. doi: 10.1093/clinids/21.1.196.
11. Uff JS, Evans DJ. Mesangio-capillary glomerulonephritis associated with Q-fever endocarditis. Histopathology. 1977 Nov;1(6):463-72. doi: 10.1111/j.1365-2559.1977.tb01685.x.

12. Leclerc S, Royal V, Dufresne SF, Mondésert B, Laurin LP. Membranoproliferative Glomerulonephritis in a Patient With Chronic Q Fever. Kidney Int Rep. 2020 Oct 8;5(12):2393-2398. doi: 10.1016/j.ekir.2020.09.038.
13. Peric L, Sabadi D, Rubil I, Bogdan M, Guzvinec M, Dakovic Rode O, et al. Imported brucellosis and Q-fever coinfection in Croatia: a case report. J Infect Dev Ctries. 2018 Jun 30;12(6):499-503. doi: 10.3855/jidc.10151.
14. Wilson MR, Naccache SN, Samayoa E, Biagtan M, Bashir H, Yu G, Salamat SM, et al. Actionable diagnosis of neuroleptospirosis by next-generation sequencing. N Engl J Med. 2014 Jun 19;370(25):2408-17. doi: 10.1056/NEJMoa1401268. Epub 2014 Jun 4.
15. Tsang CC, Teng JLL, Lau SKP, Woo PCY. Rapid Genomic Diagnosis of Fungal Infections in the Age of Next-Generation Sequencing. J Fungi (Basel). 2021 Aug 5;7(8):636. doi: 10.3390/jof7080636. PMID: 34436175.
16. Xing F, Hung DL, Lo SKF, Chen S, Lau SKP, Woo PCY. Next-generation sequencing-based diagnosis of bacteremic Listeria monocytogenes meningitis in a patient with anti-interferon gamma autoantibodies: a case report. Infect Microb Dis. Forthcoming.
17. Xing F, Lo SKF, Ma Y, Ip JD, Chan WM, Zhou M, et al. Rapid diagnosis of Mycobacterium marinum infection by next-generation sequencing: a case report. Front Med (Lausanne). Forthcoming.
18. Kondo M, Dalai SC, Venkatasubrahmanyam S, Eisenberg N, Robinson BD, Westblade LF, et al. Diagnosis and Genotyping of *Coxiella burnetii* Endocarditis in a Patient with Prosthetic Pulmonary Valve Replacement Using Next-Generation Sequencing of Plasma Microbial Cell-Free DNA. Open Forum Infect Dis. 2019 Jun 1;6(6):ofz242. doi: 10.1093/ofid/ofz242.
19. Huang M, Ma J, Jiao J, Li C, Chen L, Zhu Z, et al. The epidemic of Q fever in 2018 to 2019 in Zhuhai city of China determined by metagenomic next-generation sequencing.

PLoS Negl Trop Dis. 2021 Jul 15;15(7):e0009520. doi: 10.1371/journal.pntd.0009520.

20. Huang J, Wang R, Gao C, Lü Y, Cao Z, Deng S, et al. A case of tick-transmitted Q fever in Lishui, China diagnosed by next-generation sequencing. J Int Med Res. 2021 Sep;49(9):3000605211025398. doi: 10.1177/03000605211025398.

Supporting information

S1 Table. Primers and probe for *Coxiella burnetii IS*1111 gene nested real-time PCR

S2 Table. Master mix for *Coxiella burnetii IS*1111 gene nested real-time PCR

S3 Table. Cycling profile of *Coxiella burnetii IS*1111 gene nested real-time PCR

Fig 1

Click here to access/download;Figure;Fig 1.tif

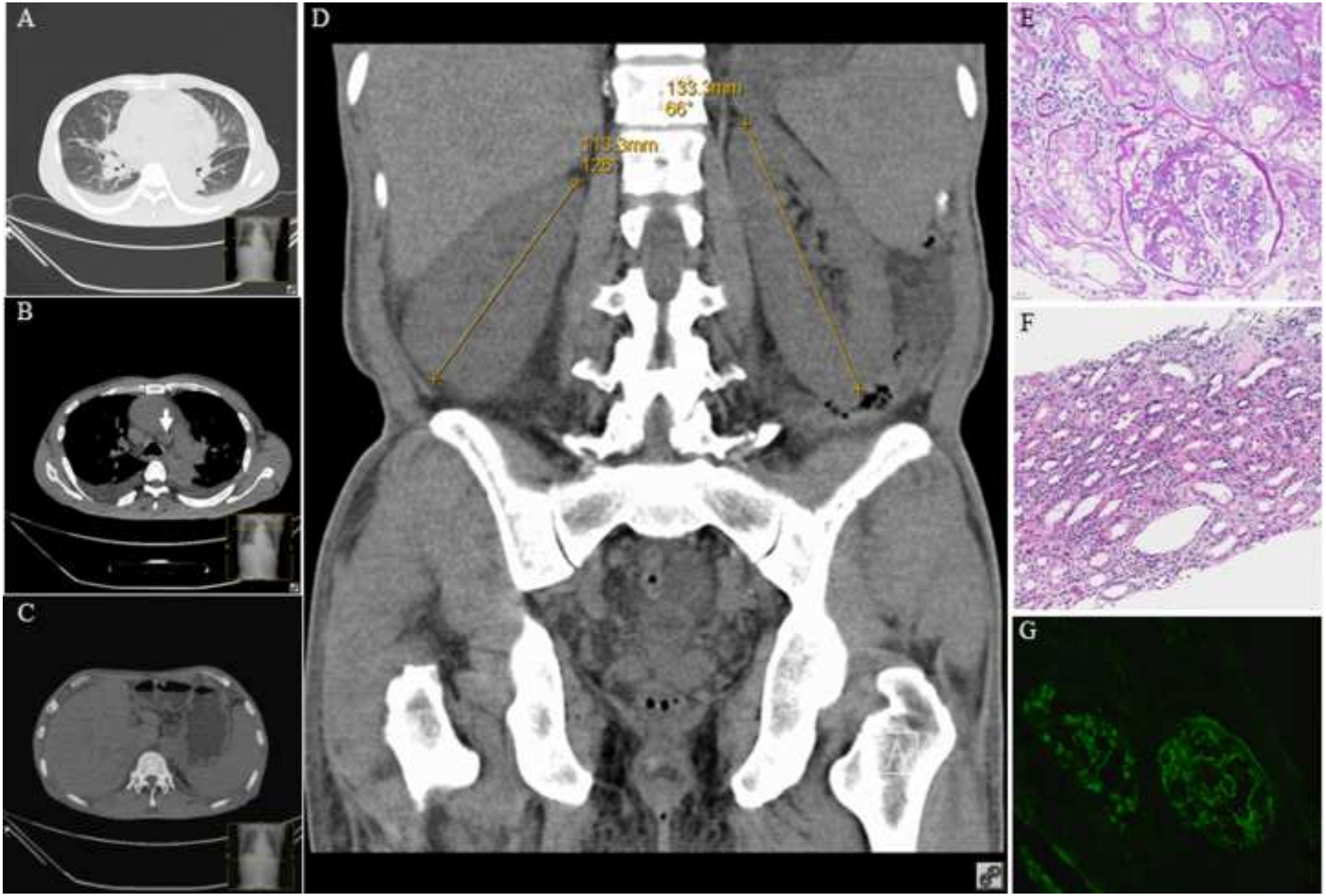

Fig 2

Click here to access/download;Figure;Fig 2.tif

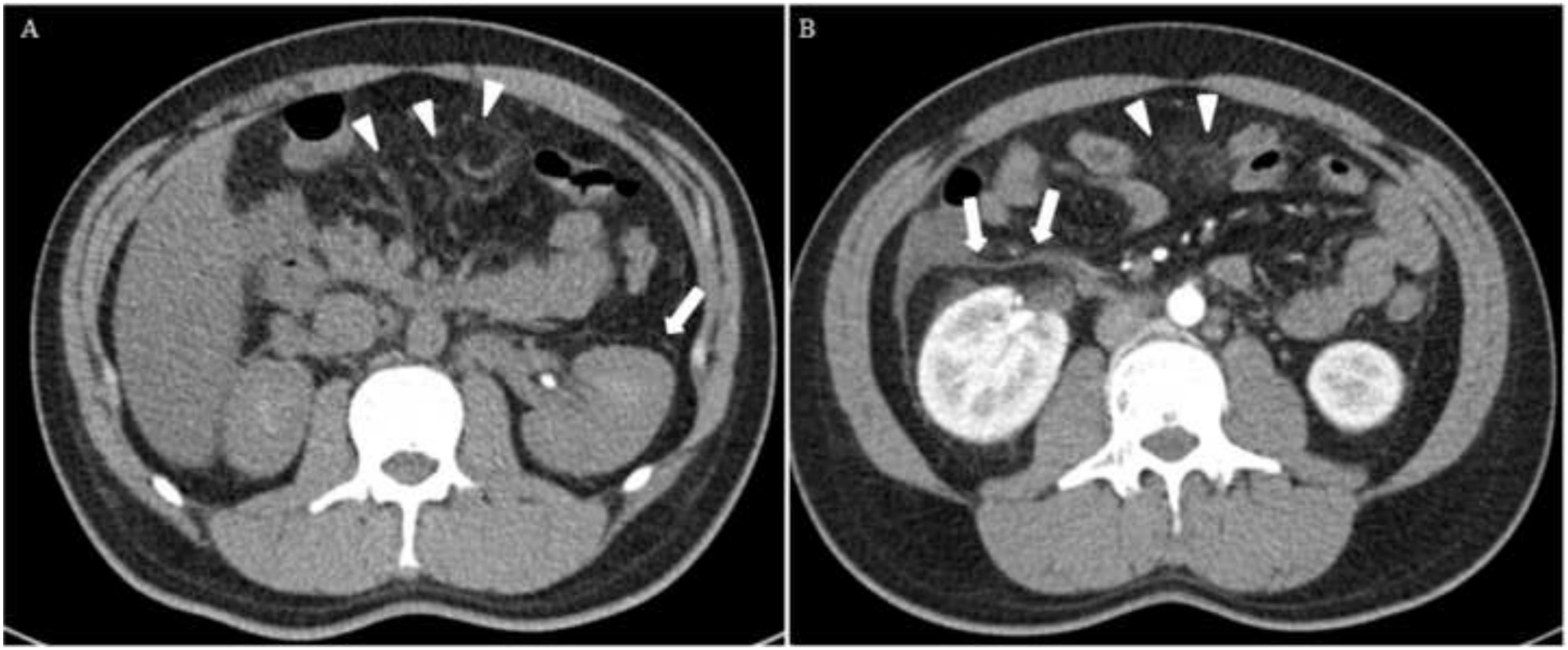

S1_Table

Click here to access/download
Supporting Information
S1_Table.docx

S2_Table

Click here to access/download
Supporting Information
S2_Table.docx

S3_Table

Click here to access/download
Supporting Information
S3_Table.docx
