# Supplementary material for: Diverse and atypical manifestations of Q fever in a metropolitan city hospital: Emerging role of next-generation sequencing for laboratory diagnosis of Coxiella burnetii
Source: PLoS Negl Trop Dis. 2022 Apr 20;16(4):e0010364. doi: 10.1371/journal.pntd.0010364 (PMC9060374; doi:10.1371/journal.pntd.0010364)
Supplement: Attachment — Submitted filename: letter of Response to comments.docx [file pntd.0010364.s005.docx]

22 March, 2022

Dear Editor,

Thanks very much for you and all the reviewers.

The contents below are the responses to the review comments and a description of the changes made in the manuscript.

**Summary and General Comments**
**Reviewer #1:**

In this work, the authors describe the symptoms of Q-fever in 14 cases from a southern Chinese city. This work contributes to our understanding of Q-fever symptoms. The title and some discussion in the text seem to suggest that next gen sequencing played a major role in this study and evidence will be presented about its utility when traditional methods fail. This however is not the case and I would suggest removal of this aspect from the title and some of the discussion. I am not advocating that the authors remove the use of next gen sequencing in their methods – it just appears to be over-hyped in the discussion. There are various important aspects of the methods (biological samples used for DNA analyses, and sequencing details) that are missing and thus make it difficult to evaluate this work. Overall, the report is well written, but certain minor English problems are prevalent. Also, in some cases, sentences contain a mixture of seemingly unrelated concepts that make it difficult to precisely identify the point being made by the authors.

**Response: In this study, the various laboratory tests were used for confirmation of the diagnosis of Q fever. When the clinical features and epidemiology of the patients were analyzed, it was observed that there were a diverse and atypical manifestations of Q fever in the cohort, which is the primary message of this paper. As for the laboratory tests themselves, serology and PCR have been widely used for the diagnosis of Q fever. However, NGS was not commonly used and reported in the literature. Since this emerging technology in clinical microbiology was used successfully for the diagnosis of Q fever in our patients, this is another important message that we would like to emphasize, not only for Q fever but also for other culture-negative infections, whereas Q fever is an example. Therefore, we think that this is an important point that we would like to make and hence include it in the title as a secondary message as well as the discussion. However, if the reviewer and editor insist that this should be removed from the title, we can make further revision. Yet, we have followed the reviewer’s suggestion to remove Cb is a potential biological weapon from the title.**

**As for the details in methodology, we have responded the reviewer’s comments in our subsequent responses.**

Q fever is thought to be underdiagnosed throughout the world due to the diversity of non-specific symptoms. What is different about these cases compared to previously reported symptoms. Authors mention “atypical” manifestations, but the reader has to get to the discussion (line 225) to find the first comparison to existing literature.

**Response: In the Introduction, “… including endocarditis, hepatitis, meningitis, encephalitis, osteomyelitis, etc” is added to give a better introduction to the diverse manifestations of Q fever.**

Authors discuss the patients and source of their data under “Patients” (Materials and Methods), and this seems to only include electronic records. Where did the material used for serological and DNA-based testing come from? Perhaps some of the results were in electronic records, but what about the material that was processed in the author’s laboratory?

**Response: The indirect immunofluorescence assay and nested real-time PCR that were performed in our laboratory as well that the NGS analysis that was sent out to a private laboratory were part of the clinical management of the patients and were also available in the electronic records. No additional test was performed for this particular study. In this revised manuscript, lines 106-108, we have added “Clinical specimens, including the sera for indirect immunofluorescence assay and blood samples for nested real-time PCR and NGS analysis, were collected and handled according to standard protocols” to reference the methods for sample collection.**

Why did the authors use IgM instead of IgG as IgM is more less specific and prone to false positives?

**Response: In mainland China, not all reagents are readily available. At the time when the laboratory started serological diagnosis of Q fever, only reagents for testing IgM was available in the market.**

The IS1111 region is a great target for detection and multiple assays have been designed. Authors do not cite what assay they are using. This is important as previous work should have been performed to validate the assay and determine sensitivity/specificity.

**Response: A reference is added for the method of performing the IS1111 nested real-time PCR assay.**

The use of next-gen sequencing needs elaboration. What sequencing platform was used, what kit was used, what sequencing depth was obtained? Bioinformatic methods are also completely missing - methods for filtering human sequencing, methods for identifying pathogen sequences, methods of ensuring that putative pathogen sequences are specific to Cb. Results on next gen sequencing are also missing – what sequences were obtained, what were the quality of the reads, what depth and breadth of Cb coverage was obtained? Including simply the number of reads that can be attributed to Cb is just not enough to convince anyone that they are from Cb – especially if the highly sensitive IS1111 pcr was negative. How do we know that these reads are not from bacterial amplification clones, adaptors, or other artificial sequence?

**Response: NGS was not performed in our laboratory, but the samples were sent out to a private laboratory for NGS and analysis, as stated in the methods section (lines 138-140), “… sent to the BGI PathoGenesis Pharmaceutical Technology Co., Ltd (Shenzhen, China) for NGS analysis …”. What we received were just laboratory reports on the number of sequences from which microorganism was detected, without the raw sequence data.**

Was serology, pcr, and sequencing performed on each sample (are only positive results shown)?

**Response: No. Different laboratory tests were performed in different patients. What was performed for a specific patient depends on what tests were available at that time and what test was requested by the clinician/microbiologist. In fact, not all tests were available throughout the study period. For example, as stated in the methods line 114-118, “Q fever serology was performed in our laboratory since September 2020 using the indirect immunofluorescence assay …” and line 127-129, “Nested real-time PCR for *C. burnetii* was performed in our laboratory since August 2021…”.**

The figures are not referenced in results section of the text (only in the discussion).

**Response: The figures are referenced in the results section, Fig. 1 at line 156 and Fig. 2 at line 157.**

Specific comments:
Title: the fact that Cb is a potential biological weapon has no bearing on this work and should be removed from the title.

**Response: This is removed from the title.**

Abstract:
Line 34: Rather than “typical occupation and exposure history”, do the authors mean “typical symptoms”?

**Response: The statement is changed to “typical clinical features”.**

Line 35: Should be in the past tense – change “have” to “had”.

**Response: It is changed to “had”.**

Line 41: Is this for the initial diagnosis? Was PCR and sequencing done on all patients?

**Response: No. Different laboratory tests were performed in different patients. What was performed for a specific patient depends on what tests were available at that time and what test was requested by the clinician/microbiologist. In fact, not all tests were available throughout the study period. As stated in the methods line 127-129, “Nested real-time PCR for *C. burnetii* was performed in our laboratory since August 2021…”.**

Line 42: This sentence needs to be clarified. I don’t think that the authors are contending that the disease was self-limiting because of atypical manifestations (as the sentence currently reads). There are a lot of concepts tied into this sentence – there are aspects of Q that cause incidence rates to be underestimated – and there is the treatment and duration of the disease. Can these concepts be separated?

**Response: The original statement is modified to “Diverse and atypical manifestations are associated with Q fever. The incidence of Q fever is likely to be underestimated” to separate the concepts.**

Line 46 (and line 60 and 90): Why is “culture-negative infections” included here as Cb is extremely difficult and time consuming to culture and probably not attempted in clinics for routine diagnosis? Do authors intend to refer to bacteria that cannot be cultured?

**Response: Culture-negative infections is a term used commonly in clinical medicine and infectious disease to refer to diagnosis not readily made by routine aerobic and anaerobic bacterial culture, which include infections caused by partially treated pyogenic bacteria, bacteria that are not routinely cultured (e.g. C. burnetii, mycoplasma), molds, and viruses.**

Line 58: What is a high “index of suspicion”?

**Response: High index of suspicion is a term used commonly in clinical medicine. It means the doctor has to be aware of the disease and readily suspect that the disease is one of the differential diagnosis of the patient, so that the appropriate investigations are performed and the diagnosis will not be missed.**

Line 70: Ingestion is not a “major route of transmission” for Q.

**Response: The phrase is modified to “… and less commonly by handling and ingestion of infected meat and milk”.**

Line 122: Change “great” to “greater”.

**Response: Great is changed to greater.**

Line 145 (and throughout): Make “case” plural when appropriate.

**Response: Case is changed to cases when appropriate.**

Line 213 and 214: I don’t understand why authors contrast typical exposure (to animals) with atypical manifestations.

**Response: The phrase is changed to “At the same time, …” to make it not a contrast to the statement before it.**

**Reviewer #2:** The manuscript is excellent and should be published

Yours sincerely,

Fanfan Xing

(On behalf of Prof Patrick Woo)
